# Functional and Evolutionary Role of Reproductive Hormonal Dysregulation Following Dietary Exposure to Singed Meat

**DOI:** 10.3390/ijms26199774

**Published:** 2025-10-08

**Authors:** Prosper Manu Abdulai, Orish Ebere Orisakwe, Costantino Parisi, Rubina Vangone, Corrado Pane, Emidio M. Sivieri, Domenico Pirozzi, Giulia Guerriero

**Affiliations:** 1 Department of Public Health Education, Faculty of Environment and Health Education, Akenten Appiah-Menka University of Skills Training and Entrepreneurial Development, Asante Mampong P.O. Box 40, Ghana; pabdulaimanu@gmail.com; 2 Advanced Research Centre, European University of Lefke, TR-10, Mersin 99780, Turkey; orish.orisakwe@uniport.edu.ng; 3 Laboratory of Comparative Endocrinology (EClab), Department of Biology, University of Naples “Federico II”, 80126 Naples, Italy; parisi.eclab@gmail.com (C.P.); vangone.eclab@gmail.com (R.V.); pane.eclab@gmail.com (C.P.); sivieri.eclab@gmail.com (E.M.S.); 4 Laboratory of Biochemical Engineering, Department of Chemical Engineering, Materials and Industrial Production (DICMaPI), University of Naples “Federico II”, 80125 Naples, Italy; dpirozzi@unina.it; 5 Interdepartmental Research Center for Environment, IRCEnv (C.I.R.AM.), University of Naples “Federico II”, 80134 Naples, Italy

**Keywords:** combustion toxicants, endocrine disruption, food safety, heavy metals, hypothalamic–pituitary–gonadal (HPG) axis, oxidative stress, reproductive hormones, reproductive toxicity

## Abstract

Consumption of meat singed with non-standard fuels is a common practice in many low- and middle-income settings, yet it may introduce combustion-derived toxicants with serious health consequences. While the toxicological effects of pollutants such as polycyclic aromatic hydrocarbons and heavy metals are well documented, the specific impact of singed meat consumption on endocrine regulation remains poorly understood. Of particular concern is the reproductive hormonal network, which not only serves as a sensitive biomarker of systemic disruption but also represents an evolutionary safeguard of fertility and generational continuity. Our study addresses this knowledge gap using male Wistar rats fed for 90 days (week 0 to week 12) on diets containing increasing proportions (25%, 50%, 75%) of meat singed with firewood, liquefied petroleum gas (LPG), or tyres. Firewood- and tyre-singed meat induced dose- and source-dependent toxicity, including hepatocellular injury, impaired protein metabolism, elevated blood urea nitrogen and creatinine, organ hypertrophy, and pronounced oxidative stress. Hormonal analysis revealed reduced testosterone alongside increased FSH, LH, and prolactin, indicating hypothalamic–pituitary–gonadal axis disruption and reproductive risk. In contrast, LPG-singed meat caused only minor alterations. These findings highlight reproductive hormones as sensitive biomarkers, underscoring the health risks of singeing practices and their evolutionary implications for fertility and population fitness.

## 1. Introduction

The practice of singeing slaughtered animal carcasses using open flame is widespread in many low and middle income countries, particularly in sub Saharan Africa, where it serves both traditional and practical roles in food processing [1,2]. While liquefied petroleum gas (LPG) is a commonly used fuel source, economic and accessibility constraints often lead to the use of alternative fuel sources such as firewood and, more concerningly, used automobile tyres. These materials differ significantly in their combustion profiles and are known to emit varying levels of toxic byproducts, including polycyclic aromatic hydrocarbons (PAHs), heavy metals, dioxins, and furans—many of which are recognized as environmental and foodborne contaminants with established health risks [3,4]. Numerous studies have reported that ingestion of food contaminated with combustion derived residues may result in oxidative stress, inflammation, and organ specific toxicities, particularly affecting the liver and kidneys—the two primary organs involved in detoxification and excretion [5,6,7,8]. Given their high perfusion rates and metabolic activity, these organs are physiologically predisposed to xenobiotic accumulation and bioactivation, making them key targets in toxicological investigations [9,10,11,12,13,14,15]. Standard biomarkers such as alanine aminotransferase (ALT), aspartate aminotransferase (AST), blood urea nitrogen (BUN), and creatinine are widely employed to assess functional integrity and are often used as first-line indicators of systemic toxicity [7,8].

Beyond these well-established targets, an increasing body of evidence points to the endocrine system as an equally sensitive and vulnerable site of disruption following exposure to combustion-derived toxicants [16,17]. Among endocrine pathways, the reproductive hormonal network is especially critical: it finely regulates gametogenesis, steroidogenesis, and sexual maturation [18,19,20,21,22], while also serving as an adaptive mechanism shaped by evolution to safeguard reproductive success under variable ecological and nutritional conditions [21,22]. From this evolutionary perspective, reproductive hormones are far more than simple biochemical messengers: they are central guardians of fertility and generational continuity, ensuring population fitness over time. This dual dimension—immediate functional regulation and long-term evolutionary safeguarding—makes reproductive hormones uniquely valuable in toxicological research. They not only provide sensitive, early biomarkers of systemic disruption, but also offer a window into mechanisms that have been conserved across mammalian evolution, linking present exposures to future reproductive health. Altered serum levels of reproductive hormones can indicate direct gonadal toxicity, pituitary dysfunction, or feedback disturbances resulting from hepatic or renal impairment of hormone metabolism and clearance [23]. Advances in plasma biomarker analysis now allow the measurement of hormone levels as minimally invasive endpoints, thereby enabling longitudinal studies within the same animals and significantly reducing the number of model animals required for comprehensive toxicological assessment [24,25,26,27]. Despite the well documented endocrine disrupting potential of PAHs and heavy metals [25,28,29,30], the impact of consuming singed meat on hormonal homeostasis remains poorly characterized.

In this context, our study investigates the effects of dietary exposure to meat singed with firewood, LPG, and used tyres in male Wistar rats. Only male rats were used to minimize the confounding effects of hormonal fluctuations associated with the estrous cycle in females, which may significantly influence metabolism and toxicological responses. Defined mixtures of singed meat were incorporated into standard chow at graded inclusion levels (25%, 50%, 75%) over a controlled exposure period. Key endpoints included liver and kidney function, indices of oxidative stress, and reproductive hormone profiles, allowing us to assess dose- and source-dependent toxicity. We hypothesized that dietary exposure to meat singed with firewood and used tyres would induce systemic toxicity in a dose- and source-dependent manner—manifested as oxidative stress, hepatorenal dysfunction, and disruption of reproductive hormones—whereas LPG-singed meat would exert comparatively minor effects. By integrating hormonal biomarkers, this work aims not only to clarify the systemic risks of poorly regulated meat processing practices but also to highlight the functional and evolutionary significance of reproductive endocrine disruption.

## 2. Results

### 2.1. Effect of Consumption of Singed Meat on Body Weight in Rats over a 12-Week Exposure Period

**Figure 1:**
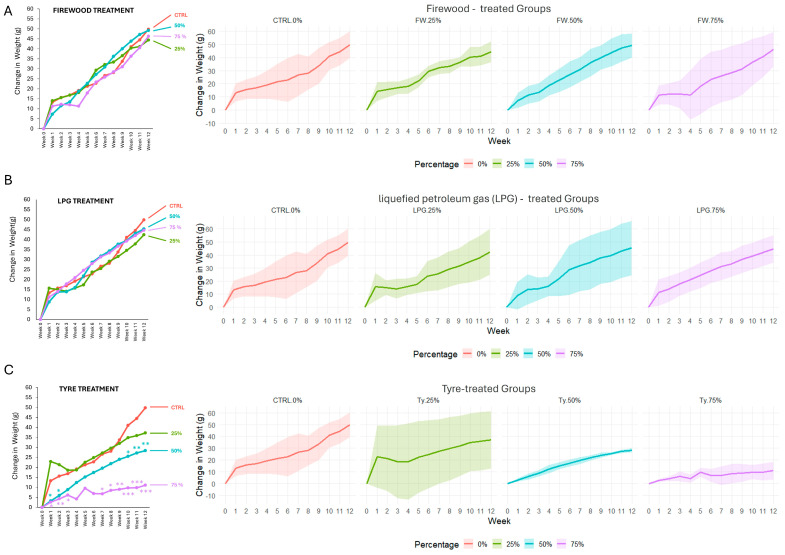
Effect of consumption of meat singed with different fuel sources ((**A**) Firewood treatment; (**B**) liquefied petroleum gas, LPO Treatment; (**C**) TYRE Treatment) and exposure levels on body weight gain in rats over a 12 week exposure period. Data points are mean ± SD (shaded areas). * *p* < 0.01, ** *p* < 0.05, *** *p* < 0.001 = statistically significant differences from control.

Body weight change was monitored weekly over a 12-week period to evaluate the potential toxicity of consuming meat singed with Firewood, LPG, and Tyre (Figure 1). Rats were grouped based on the source of singeing and were further subdivided into 25%, 50%, and 75% exposure *levels relative to the meat content in their diet.*

A control group receiving unsinged meat was included for baseline comparison. Body weight trends reveal differential toxicity profiles among the three singeing methods. In the firewood-treated groups, none of the exposure subgroups (25%, 50%, and 75%) exhibited statistically significant weekly body weight gain at any point compared to the control group (Figure 1A). Notably, the 75% firewood group showed broader standard deviation bands, especially from the fourth week, suggesting greater individual variability in response to the highest dose.

Similarly, the LPG-treated groups did not show any significant changes in weekly body weight gain under any exposure condition compared to the control (Figure 1B). The 50% LPG group exhibited broader individual variability, particularly from the sixth week onward. The tyre-singed meat groups showed a consistent reduction in weekly body weight gain for the 50% and 75% exposure groups (Figure 1C). Specifically, the 75% exposure group displayed the most pronounced effect, with significantly lower body weights compared to control across the entire experimental duration, along with a visibly flattened growth trajectory. The 50% tyre group also showed a marked reduction in weight gain for the entire study period with significantly lower weight gain at weeks 1 and 2, and again at weeks 10, 11, and 12, (* *p* < 0.05 and ** *p* < 0.01), and similarly exhibited a flattened growth trajectory and reduced variation. In contrast, the 25% tyre group displayed higher weight gain at weeks 1 and 2 and lower weight gain at weeks 10, 11 and 12 but without reaching statistical significance. It is worthwhile to observe that at weeks 0–1, the 25% group initially gained weight at a steeper slope than the control group, with a wider range and broader standard deviation, suggesting greater individual variability.

### 2.2. Body Weight at Endpoint

The final body weight gained at the study’s conclusion (week 12) summarizes the longitudinal growth trends observed over the experimental period and allows a more definitive comparison of body weight gain across the experimental groups. The rats in the control group exhibited healthy weight gain over the 12-week period with a mean final weight gain of 49.8 ± 10.4 g. Groups exposed to meat singed with firewood or LPG displayed modest variations in terminal weight gain (Figure 1A,B). Specifically, rats in the firewood-treated groups showed final mean body weight gains of 44.4 ± 8.0 g, 49.2 ± 9.3 g, and 46.3 ± 13.2 g for the 25%, 50%, and 75% groups, respectively. Similarly, LPG-treated groups recorded final average body weight gains of 42.4 ± 17.7 g (25%), 45.3 ± 20.9 g (50%), and 44.7 ± 10.5 g (75%). Body weight gains at 12 weeks for both the firewood and LPG exposed groups were similar to gains in the control group and did not demonstrate dose dependency. These results suggest that dietary exposure to firewood- and LPG-singed meat did not significantly impede overall growth or induce severe systemic toxicity at the tested concentrations.

Conversely, a markedly different trend was observed in animals exposed to tyre-singed meat (Figure 1C). The 25% tyre group presented with a mean terminal weight gain of 37.3 ± 24.3 g. The 50% tyre group experienced a further significant reduction in terminal body weight gain of 28.4 ± 1.9 g compared to control (*p* < 0.01) with a relatively narrow standard deviation suggesting a consistent negative effect at this dose. Strikingly, rats in the 75% tyre group displayed the most profound lower weight gain compared to control, with a group mean of only 11.1 ± 7.5 g at 12 weeks (*p* < 0.001). The clear dose-dependent decrease in body weight gain observed in tyre-treated groups strongly suggests that consumption of tyre-singed meat exerts detrimental effects on growth and general health.

### 2.3. Liver Function Test

**Figure 2:**
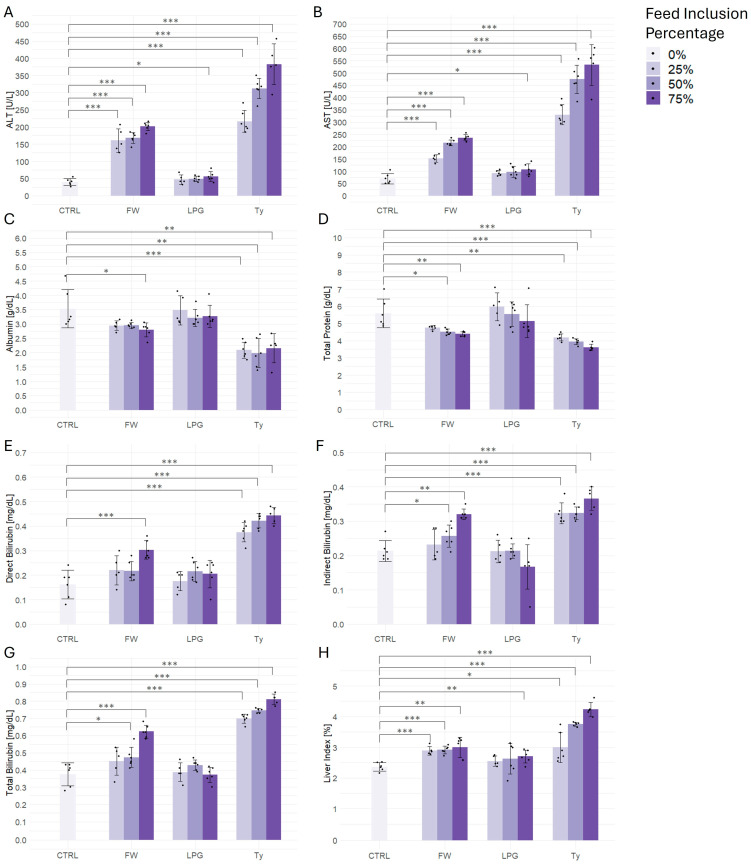
Effect of firewood- (FW), liquefied petroleum gas- (LPG), and tyre (Ty)-singed meat on liver function biomarkers in rats. (**A**) Alanine aminotransferase (ALT), (**B**) Aspartate aminotransferase (AST), (**C**) Serum albumin, (**D**) Total protein, (**E**) Direct bilirubin, (**F**) Indirect bilirubin, (**G**) Total bilirubin, and (**H**) Liver index was measured in rats after exposure to 0%, 25%, 50%, and 75% inclusion levels of singed meat over the experimental period. Data are presented as mean ± SD (n = 6 per group), with individual data points shown for each test subject Asterisks indicate statistical significance compared to the control group (* *p* < 0.05, ** *p* < 0.01, *** *p* < 0.001).

Biochemical markers of hepatic function revealed marked alterations in response to the consumption of singed meat, particularly in animals treated with firewood and tyre-processed samples (Figure 2). Transaminase activity, including ALT (Figure 2A) and AST (Figure 2B), showed strong, dose-dependent elevations in the firewood and tyre-treated groups. Tyre-exposed rats exhibited the highest increases, with ALT and AST levels rising approximately six- to nine-fold compared to control at the 75% inclusion level. Firewood-treated groups also demonstrated significant increases at all tested concentrations, while LPG-treated groups remained statistically similar to controls except for the 75% inclusion dose. Serum albumin (Figure 2C) showed a modest but significant reduction at all inclusion levels in the tyre group compared to control, whereas firewood and LPG groups remained largely unchanged. Total protein levels (Figure 2D) were similarly reduced in the tyre group, with significant differences observed at all three inclusion levels, while firewood-exposed rats also showed a dose-dependent decline. No significant changes were observed in the LPG groups. Bilirubin metabolism was notably impacted by tyre exposure. Direct bilirubin (Figure 2E), indirect bilirubin (Figure 2F), and total bilirubin (Figure 2G) levels were significantly elevated in a dose-dependent fashion in tyre-treated rats, with moderate, but significant, increases also observed in firewood-treated groups at higher inclusion levels. In contrast, LPG-treated groups showed no significant alterations across all bilirubin measures. Finally, the liver index (Figure 2H), representing liver weight relative to body weight, was significantly elevated in tyre-treated rats at all inclusion levels. Likewise, firewood-treated groups showed moderate but significant increases at all inclusion levels also, while LPG treatment resulted in a significant increase only at the 75% treatment level.

### 2.4. Liver Oxidative Stress

**Figure 3:**
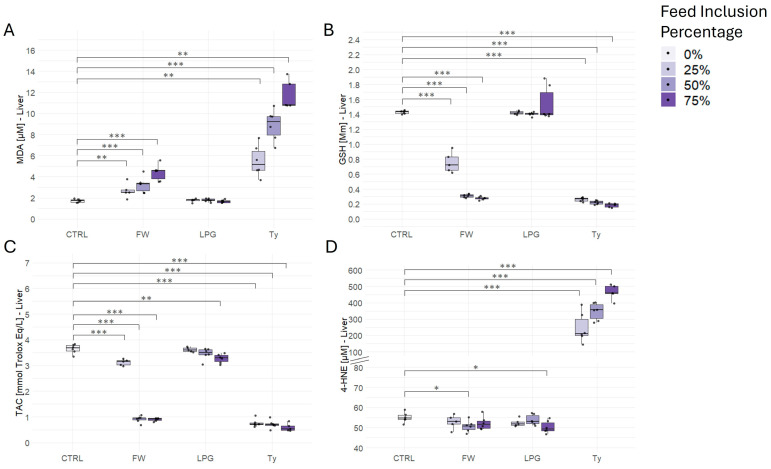
Effect of firewood- (FW), liquefied petroleum gas- (LPG), and tyre (Ty)-singed meat on hepatic oxidative stress. (**A**) Malondialdehyde (MDA), (**B**) reduced glutathione (GSH), (**C**) total antioxidant capacity (TAC), and (**D**) 4-hydroxynonenal (4-HNE) levels were measured in liver tissues of rats fed standard chow (CTRL) or chow containing 25%, 50%, or 75% inclusion levels of FW, LPG, and Ty singed meat. Data are presented as mean ± SD (n = 6), with individual data points shown for each test subject. Statistical significance is indicated: * *p* < 0.05, ** *p* < 0.01, *** *p* < 0.001.

Rats exposed to firewood- and tyre-singed meat exhibited significantly increased hepatic malondialdehyde (MDA) levels compared to the control group, indicating enhanced lipid peroxidation (Figure 3A). A clear dose-dependent trend was observed in both firewood- and tyre-treated groups, with the 75% tyre group showing the highest MDA levels. In contrast, LPG-treated groups did not differ significantly from the control at any inclusion level.

Regarding hepatic glutathione (GSH) levels, rats fed firewood- and tyre-singed meat displayed a significant and progressive reduction compared to the control (Figure 3B). The dose-dependent reduction was more marked in the tyre-treated groups, with values approaching depletion at higher inclusion levels. Conversely, GSH levels in LPG-treated rats remained comparable to the control, showing no significant changes.

Total antioxidant capacity (TAC) in the liver was significantly diminished in rats exposed to firewood- and tyre-singed meat in a dose-dependent manner, further reflecting oxidative stress (Figure 3C). LPG-treated groups showed no significant difference in TAC at 25% and 50% inclusion levels compared to the control, supporting a relatively stable redox status in these animals.

For 4-hydroxynonenal (4-HNE), a marker of oxidative lipid damage, rats fed tyre-singed meat showed a striking and significant increase at all inclusion levels (Figure 3D). A modest but significant elevation was also observed at the 50% and 75% inclusion levels in firewood- and LPG-singed meat groups, respectively.

### 2.5. Kidney Function Test

**Figure 4:**
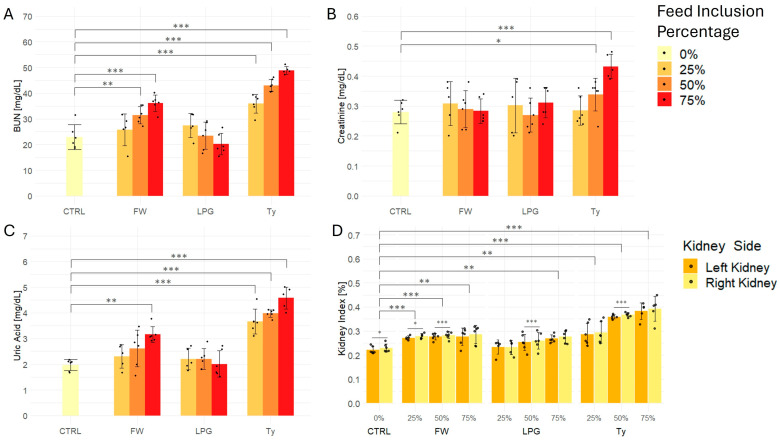
Effect of firewood- (FW), liquefied petroleum gas- (LPG), and tyre (Ty)-singed meat on kidney function biomarkers and kidney index in rats. (**A**) Blood urea nitrogen (BUN), (**B**) creatinine, and (**C**) uric acid levels, along with (**D**) kidney index (left and right kidneys shown separately), were evaluated in rats fed diets containing 0% (control), 25%, 50%, or 75% inclusion of meat singed using firewood (FW), liquefied petroleum gas (LPG), or used tyres (Ty). Data are presented as mean ± SD (n = 6 per group), with individual data points shown for each test subject. Statistical significance is indicated as: * *p* < 0.05, ** *p* < 0.01, *** *p* < 0.001.

Rats exposed to firewood- and tyre-singed meat exhibited significantly increased BUN levels compared to the control group, with a clear dose-dependent trend observed particularly in the tyre-treated groups (Figure 4A). Notably, rats in the 75% tyre group showed the highest BUN concentrations among all treatment groups. LPG-treated groups did not show significant alterations in BUN levels compared to control.

Regarding serum creatinine, rats exposed to the 50% and 75% tyre-singed meat displayed significantly elevated levels relative to the control. No significant differences were observed in the firewood- or LPG-treated groups across all percentage levels (Figure 4B).

Rats fed tyre-singed meat also exhibited significantly elevated serum uric acid levels in a dose-dependent manner compared to control, with the highest values recorded in the 75% group (Figure 4C). In contrast, uric acid levels in rats in the firewood treated groups at the 25% and 50% inclusion levels were not significantly different from control. Likewise, uric acid levels in LPG treated rats across all inclusion levels were not significantly different from control.

Concerning the kidney index, interestingly, within the control group, the left and right kidneys differed significantly, suggesting that this variation may be physiological. Additionally, significant differences between the left and right kidneys were observed in some treated groups, including firewood at 25% and 50% inclusion levels, LPG at 50%, and tyre at 50% inclusion level (Figure 4D).

Considering kidney index values from the averaged left and right kidneys, rats in the firewood- and tyre-treated groups demonstrated a significant increase in kidney index compared to the control, showing a clear dose-dependent trend (Figure 4D). The 75% tyre-treated group exhibited the most pronounced increase. The LPG-treated group showed a modest but significant elevation in kidney index only at the 75% inclusion level.

### 2.6. Reproductive Hormones Profile

The evaluation of serum reproductive hormone plasma levels revealed marked disruptions in the hypothalamic–pituitary–gonadal (HPG) axis among rats particularly exposed to meat singed with firewood and, more notably, used tyres.

**Figure 5:**
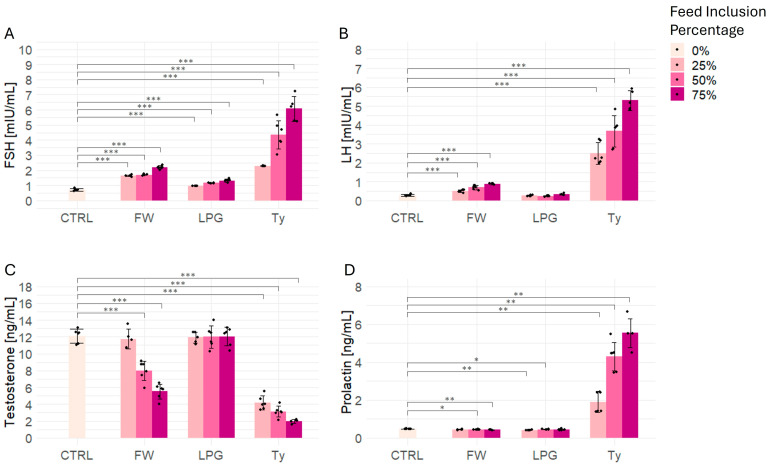
Effect of firewood- (FW), liquefied petroleum gas- (LPG), and tyre (Ty)-singed meat on reproductive hormone levels in male rats. (**A**) Follicle-Stimulating Hormone (FSH), (**B**) Luteinizing Hormone (LH), (**C**) Testosterone, and (**D**) Prolactin levels measured in serum following dietary exposure to meat singed with firewood, liquefied petroleum gas, or used tyres at increasing inclusion ratios (25%, 50%, 75%). Data are expressed as mean ± SEM (n = 6 per group), with individual data points shown for each test subject. Statistical significance relative to the control group (CTRL): * *p* < 0.05, ** *p* < 0.01, *** *p* < 0.001.

As shown in Figure 5A, FSH levels increased significantly and dose-dependently in the FW, LPG and Ty groups compared to the control. Particularly, tyre-treated rats displayed the most dramatic increase. In the FW and LPG group, significant increases were also observed at all inclusion levels, although to a lesser extent than in the Ty group.

A similar pattern was observed for LH (Figure 5B), with a dose-dependent increase most evident in the Ty group, reaching nearly twenty-fold higher levels than controls. The FW group also showed significantly elevated LH values at all inclusion levels. In contrast, the LPG group at had insignificant increases in LH at all inclusion levels.

Conversely, testosterone concentrations (Figure 5C) showed marked significant reductions in both the FW and Ty groups in a dose-responsive manner. Tyre exposure led to a dramatic suppression of testosterone, with levels falling below 4 ng/mL. Similarly, FW-treated animals showed a significant testosterone decline at 50% and 75% inclusion, though the reduction was slightly less severe than in the Ty group. Interestingly, testosterone levels in the LPG group were not significantly altered, indicating preserved Leydig cell function and endocrine balance.

Prolactin levels were significantly elevated in Ty-treated animals, showing a robust dose-dependent increase with maximal levels exceeding 5 ng/mL at 75% inclusion (Figure 5D). FW-treated rats also exhibited significant prolactin increases at 50%, and 75% inclusion levels as well as in LPG-treated group at 25% and 50%, though these were less dramatic than in the Ty group.

### 2.7. Clinical Observations and Endpoint Criteria Evaluation

During the study, the rats in the control group and those fed LPG maintained normal posture, groomed themselves well, had good coat condition and were responsive. In contrast, animals exposed to meat burned with firewood, particularly tyres, showed reduced grooming activity and lower vitality, a trend that became more evident with higher inclusion levels. Daily monitoring revealed stable food and water intake in the control and LPG groups. Rats fed meat burned with tyres, particularly at 50% and 75%, gradually reduced their appetite in line with their reduced weight gain. Body weight increased steadily in the control and LPG groups, with minimal changes in rats fed firewood. However, the tyre-fed groups showed clear dose-dependent growth suppression, with almost zero weight gain at the 75% level. Signs such as piloerection, lethargy, reduced locomotor activity and occasional breathing difficulties or diarrhoea were mainly observed in groups treated with high doses of tyres. Animals fed firewood showed milder symptoms, while those fed LPG showed no obvious signs of toxicity. No animals met the humane endpoint criteria (e.g., weight loss of more than 20%, inability to eat or drink, or a moribund state). However, the groups fed high-dose tyres showed obvious systemic impairment, underscoring the severity of this treatment.

## 3. Discussion

### 3.1. Systemic Toxicity

The current study indicates that LPG-singed meat supported normal weight gain, unlike firewood- and tyre-singed meats, which impaired growth at higher exposures. Body weight is a vital indicator of nutritional and metabolic health [31,32]. Clean, uncontaminated food supports normal weight gain by ensuring efficient nutrient absorption, metabolism, and hormonal balance [33]. In contrast, contaminated food, often containing heavy metals, PAHs, and other toxicants, can impair these processes, leading to poor weight gain or weight loss [34,35]. Systemic toxicity can arise from the ingestion of meat processed with hazardous materials, particularly discarded tyres and unregulated firewood. Tyres are composed of a complex mixture of synthetic polymers (e.g., styrene-butadiene rubber), carbon black, plasticizers, and vulcanizing agents such as sulfur, along with stabilizers and fillers that include heavy metals like zinc, lead, cadmium, and chromium [36]. During combustion, these substances degrade and release a range of toxic by-products, including polycyclic aromatic hydrocarbons (PAHs), volatile organic compounds (VOCs), dioxins, furans, and heavy metal oxides [1,2,36,37]. Furthermore, combustion of tyres at low temperatures can lead to the formation of ultrafine particles, which are particularly bioavailable and toxic upon ingestion. Firewood, while a more traditional fuel, also generates substantial quantities of soot, carbon monoxide, PAHs, and fine particulate matter when burned inefficiently [38,39]. These combustion products can deposit on the surface of singed meat and subsequently enter the human body upon ingestion. Toxicants in food can damage the gastrointestinal lining, disrupt gut microbiota, and interfere with nutrient uptake. They also impair the liver and kidneys—key organs in metabolism and detoxification—resulting in reduced nutrient utilization. Moreover, many food-borne contaminants act as endocrine disruptors, altering hormones like insulin and leptin that regulate appetite and energy balance [5]. Additionally, contaminated food increases oxidative stress and systemic inflammation, which can cause muscle wasting and elevate energy expenditure, further suppressing weight gain and potential carcinogenic risk [1,40,41]. Experimental studies in animals have shown that exposure to food processed with harmful substances, such as tyres or smoke residues, leads to significant reductions in body weight, especially at higher exposure levels [2,42]. In contrast, food processed using safer methods like LPG singeing, which produces fewer toxic residues, supports healthier weight outcomes [43]. These findings highlight the need for public health policies that promote safe food processing practices, discourage hazardous methods, and ensure access to clean food to protect nutritional health and prevent toxicity-related growth impairments.

### 3.2. Hepatic and Renal Toxicity

In this study, it was demonstrated that consumption of meat singed with firewood or used tyres induces marked hepatorenal dysfunction and oxidative stress in Wistar rats, whereas meat singed with LPG elicits minimal toxic effects. Rats fed firewood- and tyre-singed meat exhibited dose-dependent elevations in serum markers of liver injury (ALT, AST), reductions in synthetic function (albumin, total protein), impaired bilirubin clearance, and increased liver indices. Concurrently, indices of renal dysfunction (BUN, creatinine, uric acid) and kidney index were significantly elevated in these same groups. Oxidative stress assays revealed pronounced lipid peroxidation (MDA, 4 HNE) and depletion of antioxidant defenses (GSH, TAC) in liver tissue following firewood and tyre exposure. Conversely, LPG-singed meat produced biomarker profiles comparable to controls across all assays. These results clearly show how traditional but unsafe meat processing practices can silently burden vital detoxification organs, gradually eroding their ability to maintain systemic homeostasis as already highlighted by our studies [1].

### 3.3. Combustion-Derived Toxicants

The heightened hepatotoxicity observed in tyre- and firewood-treated groups likely reflects the complex mixture of combustion-derived toxins produced during the singeing process. Automobile tyres release polycyclic aromatic hydrocarbons (PAHs), volatile organic compounds, dioxins, furans, and heavy metals upon combustion; many of these are established genotoxic and pro-oxidant agents [44,45]. Similarly, incomplete biomass combustion generates PAHs, particulate matter, and reactive free radicals that can deposit on meat surfaces and enter the organism upon ingestion [46,47]. In contrast, LPG combustion is more complete, producing primarily carbon dioxide and water vapor under optimal combustion conditions, with minimal soot or secondary toxicants [48,49]. This study aligns with previous reports linking dietary PAH ingestion to elevated hepatic transaminases and impaired renal clearance in rodent models [50,51].

### 3.4. Mechanistic Insights into Oxidative Stress

Mechanistically, PAHs and metal contaminants can initiate reactive oxygen species (ROS) formation via redox cycling or through the activation of cytochrome P450 enzymes, particularly CYP1A1, thereby triggering lipid peroxidation, protein oxidation, and DNA damage in hepatocytes and renal tubular cells [44,52]. The observed depletion of GSH—a frontline intracellular antioxidant—further compromises cellular defense, exacerbating oxidative injury [53,54]. 4-HNE, a cytotoxic aldehyde formed during lipid peroxidation, forms adduct with proteins and DNA, disrupting membrane integrity and enzyme function, and has been implicated in inflammatory signaling cascades and cell death pathways [55,56]. The current study’s findings of dramatically elevated 4 HNE and MDA levels in the tyre group thus reflect profound oxidative damage, consistent with earlier work showing that tyre-derived particulates are highly pro-oxidant [42,57]. This mechanistic chain illustrates how dietary exposure to combustion residues can overwhelm natural defenses, setting off a cascade of molecular events that damage cells and impair organ function.

### 3.5. Renal Impairment

The differential impact on kidney function likely arises both from direct tubular toxicity and from secondary hemodynamic effects. Elevated BUN and creatinine indicate reduced glomerular filtration rate, while increased uric acid may reflect both impaired excretion and heightened purine catabolism under oxidative stress [58,59,60]. The kidney index enlargement suggests hypertrophy or inflammation, possibly tied to compensatory responses to toxin overload. Prior studies have noted that PAH exposure can impair renal transporters and induce interstitial fibrosis in chronically exposed rodents [61,62].

### 3.6. Reproductive Hormonal Disruption

Our findings show that plasma hormone profiling is not only technically feasible but also highly informative. In rats exposed to firewood- and tyre-singed meat, we observed a distinct hormonal signature: testosterone levels were consistently reduced, while FSH, LH, and prolactin increased in a dose-dependent manner. These results illustrate how quickly the reproductive endocrine system responds to toxicant exposure, providing a functional readout of hypothalamic–pituitary–gonadal axis disruption that complements the evidence of liver and kidney injury. These findings align with literature showing that PAHs and dioxin-like compounds can impair steroidogenesis, damage Leydig cells, and disturb endocrine signaling through oxidative and receptor-mediated mechanisms [63,64]. The ability to detect such changes through plasma analysis offers a major methodological advantage. Unlike traditional terminal assays, hormone profiling is minimally invasive, enables longitudinal monitoring in the same animals, and aligns with the ethical principles of the 3Rs (Replacement, Reduction, and Refinement) in toxicological research [27,65]. By capturing endocrine alterations that mirrored oxidative stress and organ dysfunction, our study highlights reproductive hormones as integrative biomarkers that bridge molecular events to systemic outcomes.

### 3.7. Hormonal Biomarkers and Translational Relevance

In this study, plasma hormone profiling revealed a clear and consistent pattern of disruption: animals exposed to firewood- and tyre-singed meat showed elevated FSH, LH, and prolactin alongside suppressed testosterone, while LPG-exposed rats exhibited hormone profiles largely comparable to controls. These measurable changes provide more than simple biochemical data—they demonstrate how sensitive reproductive hormones are to environmental toxicants, reflecting early functional disturbances in the hypothalamic–pituitary–gonadal axis. Importantly, detecting these alterations through plasma analysis offers a significant methodological advantage. Plasma hormone profiling is non-lethal and minimally invasive, allowing the real-time monitoring of systemic toxicity while reducing animal euthanasia and aligning with the 3Rs principles of animal research (Replacement, Reduction, and Refinement) [27,65]. By capturing hormonal shifts that paralleled organ-specific injury in the liver and kidneys, our results strengthen the case for hormones as integrative biomarkers, capable of linking oxidative stress and organ dysfunction with reproductive health. Moreover, since these hormones can be reliably measured in humans, their use enhances the translational impact of the findings. The observed consistency between organ injury and endocrine disruption underscores the validity of reproductive hormones as sensitive early-warning indicators of toxicant exposure, with direct applicability to public health monitoring in populations at risk.

### 3.8. Functional and Evolutionary Role of Hormones

The incorporation of hormone profiling not only broadens the scope of toxicological evaluation but also points to viable, ethically responsible strategies for risk assessment and regulation in both experimental and public health contexts. In this study, rats exposed to firewood- and tyre-singed meat exhibited a clear hormonal signature of disruption, with significant elevations of FSH, LH, and prolactin, accompanied by suppressed testosterone. These changes indicate hypothalamic–pituitary–gonadal axis dysregulation and suggest impaired androgen synthesis and testicular function [21,22]. Such alterations are not merely biochemical shifts: reproductive hormones are fundamental to fertility and species continuity, orchestrating gamete production, sexual maturation, and reproductive cycles [21,22]. The imbalance observed here shows how easily this delicate system—refined through evolution to secure reproductive success—can be destabilized by environmental and dietary stressors. In this context, the endocrine disruption caused by singed meat exposure emerges as a concrete example of how common dietary practices may carry hidden risks [1,21,22]. By compromising testosterone production and stimulating compensatory gonadotropin release, toxicants from combustion residues may impair reproductive fitness. Thus, the hormonal changes detected in our results highlight both the functional role of hormones as sensitive early-warning biomarkers and their evolutionary role as guardians of fertility and generational continuity.

### 3.9. Clinical Endpoints Highlighting the Risks of Combustion-Derived Contaminants

The clinical manifestations observed in the tyre-fed groups, such as reduced cleanliness, lower vitality, loss of appetite and growth suppression in a dose-dependent manner, provide strong evidence of systemic toxicity caused by combustion-derived contaminants. These findings are consistent with reports demonstrating that exposure to tyre smoke or related pollutants induces oxidative stress, impairs organ function, and reduces physiological resilience in rats [42]. Similarly, subacute exposure to combustion particles or tyre wear debris has been associated with reduced food intake, altered body weight and organ changes, even in the absence of overt clinical toxicity [66]. In our study, no animals reached the humane endpoints (>20% weight loss, persistent inability to eat or drink, or a moribund state). However, the combination of symptoms observed in the high-dose tyre-fed groups, including lethargy, reduced locomotor activity, occasional respiratory distress and diarrhoea, clearly indicated systemic impairment. These manifestations are consistent with previous reports of dietary and particle toxicity in rodents [67]. Taken together, these observations highlight the value of clinical monitoring as a non-invasive, early indicator of toxicity that complements biochemical and histopathological findings. The stark contrast between the severe impairments in the tyre-fed groups, the milder symptoms in the firewood-fed animals and the near-normal appearance of the LPG-fed rats further supports the conclusion that burnt tyre meat poses the greatest health risk, while LPG appears to be the safest option.

## 4. Materials and Methods

### 4.1. Animal Type and Housing

Sixty Wistar male albino rats (4–6 weeks old, 28–42 days at the start of the study, with an average initial weight of 112.72 g, range 102.7–135.1 g) were purchased from Noguchi Memorial Institute for Medical Research in Accra, Ghana. The rats were kept at Kwame Nkrumah University of Science and Technology’s (KNUST) animal facility in a controlled setting. The animals were housed in sterile plastic cages, allowed to acclimatize for seven days prior to the experiment, and were kept under standard settings, which included 12 h light/dark cycles, a temperature of 25 ± 2 °C, and a humidity of 50 ± 15%. Ad libitum water and a regular rat diet were given. The University of Port Harcourt’s Research Ethics Committee granted ethical approval for the study, which complied with institutional ethical standards for animal research (UPH/CEREMAD/REC/MM73/014).

### 4.2. Study Design

Meat singed with LPG, scrap tyres, and firewood, collected from the abattoirs of Accra, Kumasi, Tamale, Koforidua, and Ho, was homogenized into powder and incorporated into a standard grower mash feed (Agricare Grower Mash, Kumasi, Ghana) in varying proportions. The control group (CTRL) received 20 g of standard rat diet per day. For the treatment groups, singed meat was mixed with chow at inclusion levels of 25%, 50%, and 75%. Specifically, rats in the firewood groups were fed diets containing 5 g, 10 g, or 15 g of firewood-singed meat balanced with 15 g, 10 g, or 5 g of standard diet, respectively. The LPG-treated groups received equivalent proportions of LPG-singed meat combined with chow, while the tyre-treated groups were similarly fed diets containing 5 g, 10 g, or 15 g of tyre-singed meat with the complementary amounts of standard diet to maintain 20 g total feed. In total, the animals were randomly assigned into ten groups (n = 6 per group): one control group and nine treatment groups exposed to firewood-, LPG-, or tyre-singed meat at 25%, 50%, or 75% inclusion levels. All animals were housed individually and maintained on their respective diets for a period of 90 days (week 0 to week 12). The exposure duration was selected in accordance with internationally recognized guidelines (e.g., OECD Test Guideline 408) for subchronic toxicity studies.

### 4.3. Sample Collection and Processing

At the end of the experimental period, animals were fasted overnight and euthanized under mild pentobarbital anaesthesia (50 mg/kg). Blood samples were collected via cardiac puncture into EDTA tubes, centrifuged at 3000 rpm for 10 min to obtain plasma, and stored at −80 °C until biochemical analysis.

### 4.4. Systemic Toxicity

#### 4.4.1. Body Weight Change Index

Body weight gain was assessed by subtracting each rat’s baseline weight (week 0) from its weight at each subsequent time point. Mean ± standard deviation (SD) of body weight gain was calculated for each group and plotted to evaluate trends over time.

#### 4.4.2. Liver and Kidney Index

Body weight, liver, and kidney were weighed at the end of the experiment using an Atom electronic balance. After the 90-day treatment period the liver and kidneys were carefully excised from each rat on a chilled dissection mat and immediately rinsed in a saline buffer solution (20 mM Tris–HCl, 0.14 M NaCl, pH 7.4) to remove blood and debris. The liver and kidney indices were calculated using the following formula:

$$\mathrm{Liver}\text{-}\mathrm{Kidney}\;\mathrm{Index}\;=\;\frac{LIver\;or\;Kidney\;weight}{Final\;rat\;body\;weight}\;\times \;100$$

#### 4.4.3. Kidney and Liver Function

Kidney function was evaluated by measuring serum creatinine, blood urea nitrogen (BUN), and electrolytes (Na^+^, K^+^, Cl^−^) using standard biochemical methods. Creatinine was assessed via the Jaffe reaction, BUN by the urease method, and electrolytes using a flame photometer and colorimetric analysis [68,69]. Acceptance criteria: drift < 5% between bracket standards; check standard within ±5% of nominal. Liver function was assessed by determining alanine aminotransferase (ALT), aspartate aminotransferase (AST), alkaline phosphatase (ALP), total bilirubin, and total protein. ALT and AST were quantified using the Reitman-Frankel method [70,71], ALP via the p-nitrophenyl phosphate (pNPP) method [72], bilirubin by the Jendrassik-Grof method, and total protein using the Biuret method [73]. Calibration/standard curves were prepared freshly on each run using manufacturer-supplied or NIST-traceable standards spanning the expected sample range (typically 5–7 non-zero levels plus blank). Linear regressions were accepted with R^2^ ≥ 0.99; back-calculated calibrators were required within ±10% of nominal (±15% at the lower limit). Two levels of internal Quality Control, QC (low/high) were run at the start and every ~20 samples; runs were repeated if QC exceeded ±2 SD of historical means. Technical triplicates were used for ALT/AST/ALP when volume allowed; otherwise duplicates. All absorbance readings were obtained spectrophotometrically, with results expressed in standard clinical units.

### 4.5. Oxidative Profile

Testis oxidative stress biomarkers including malondialdehyde (MDA), reduced glutathione (GSH), total antioxidant capacity (TAC), and 4-hydroxynonenal (4-HNE) were similarly assayed using standard colorimetric and immunochemical methods [74,75,76]. Lipid peroxidation was quantified as thiobarbituric acid reactive substances (TBARS), employing 1,1,3,3-tetramethoxypropane (TMP) as the calibration standard over a concentration range of 0–10 μM (seven points plus blank). Absorbance was read at 535–540 nm, with all samples analyzed in duplicate; standard curves were generated by ordinary least squares regression with coefficients of determination (R^2^) ≥ 0.99. Reduced glutathione was determined using the DTNB assay with a standard curve prepared from 0 to 1.0 mM reduced glutathione (seven points), also analyzed in duplicate and fitted by least-squares regression (R^2^ ≥ 0.99). Total antioxidant capacity was assessed by a Trolox-equivalent assay, with calibration standards from 0 to 2.0 mM; results were expressed as mmol Trolox equivalents. Finally, 4-HNE protein adducts were measured using a commercial ELISA kit. Each plate included a seven-point calibration curve (0–200 ng/mL), and sample duplicates were analyzed according to the manufacturer’s protocol. Curve fitting was performed with a four-parameter logistic model, and plate acceptance criteria required R^2^ ≥ 0.99, randomly distributed residuals, and a coefficient of variation ≤ 15% between technical replicates.

### 4.6. Hormonal Profile

Plasma levels of follicle-stimulating hormone (FSH), luteinizing hormone (LH), testosterone, and prolactin were determined using commercially available ELISA kits (Monobind Inc., Lake Forest, CA, USA; FSH, Cat. No. 425-300 B; LH, Cat. No. 8325-300 E (panel including LH); PRL, Cat. No. 725-300; T, Cat. No. 3725-300A) according to the methods followed by Eddie-Amadi et al. [77] and Oyewopo et al. [78], respectively. Each plate included 7-point manufacturer calibrators plus matrix-matched low/medium/high QC controls. All samples were run in technical duplicates; plates were accepted if the 4PL fit had R^2^ ≥ 0.99, calibrators/back-calculations were within ±15% of nominal (±20% at LLOQ), and duplicate %CV ≤ 15%. Inter-assay variability was monitored by repeating a pooled-plasma QC on each plate.

### 4.7. Clinical Observations and Endpoint Criteria

To assess clinical observation and endpoint criteria a summary of the parameter to evaluate was established (Table 1).

### 4.8. Biological and Technical Replication

Animals were randomized into ten groups (n = 6 rats/group: CTRL and 3 fuel sources × 3 inclusion levels), yielding six biological replicates per condition. Unless otherwise stated, each biochemical and endocrine measurement was performed in duplicate (technical duplicates) per biological sample, and the mean of the technical duplicates was used for statistical analysis. For clinical chemistry panels with automated readers, measurements were performed in technical triplicate when sample volume permitted. Figure legends already report n = 6 per group.

### 4.9. Statistical Analysis

Data are presented as mean ± standard deviation (SD). Each experimental condition was compared to the control group using a two-sample *t*-test (Student’s *t*-test) after verifying assumptions of normality and homogeneity of variance. Normality was assessed using the Shapiro–Wilk test, and equality of variances was tested using the F-test. When variances were unequal, Welch’s *t*-test was applied. For data that did not meet the assumption of normality, the non-parametric Wilcoxon rank-sum test was used.

To assess intra-animal differences in kidney weight between the right and left kidney within each condition, a paired statistical approach was used. Specifically, a paired *t*-test was applied when the distribution of the differences between paired measurements was normal (based on the Shapiro–Wilk test). If this assumption was violated, the Wilcoxon signed-rank test was used instead. A *p*-value < 0.05 was considered statistically significant. All statistical analyses were performed using R software, version 4.5.1.

## 5. Conclusions

In conclusion, our findings show that singeing meat with firewood or used tyres triggers marked liver and kidney dysfunction, oxidative stress, and clear disturbances in reproductive hormonal balance. By contrast, meat processed with LPG caused only minor changes, confirming its relative safety as a fuel source for meat preparation. Importantly, clinical observations such as reduced grooming, appetite loss, lethargy, and growth suppression in tyre-fed rats provided easily detectable, non-invasive indicators of systemic compromise, reinforcing the biochemical and hormonal evidence of toxicity. A central strength of this work lies in the use of plasma hormone profiling, a minimally invasive approach that not only refines animal experimentation but also carries strong translational relevance for human health monitoring. Hormones such as FSH, LH, testosterone, and prolactin proved to be sensitive early-warning signals of systemic disruption, underscoring their functional role in integrating organ responses and their evolutionary role in safeguarding fertility and generational continuity. Of course, further studies are required to expand these observations, including investigations in female models and detailed chemical characterization of combustion residues, which would allow stronger attribution of the observed effects to specific toxicants.

An additional limitation is the use of standard pelleted chow as the control rather than unsinged or lightly broiled meat. This choice ensured a nutritionally balanced, toxin-free, and reproducible baseline in line with OECD guidelines, but it cannot completely exclude the contribution of meat ingestion per se. Moreover, the use of untreated or thermally processed meat would have introduced additional variability at the nutritional, microbiological, and chemical levels, potentially confounding the specific effects of combustion residues. Importantly, body weight data showed no significant differences between chow-fed controls and firewood- or LPG-fed groups, while tyre-singed groups exhibited clear reductions. This pattern supports the interpretation that the observed toxic effects derive mainly from combustion residues rather than meat consumption itself. Future studies should include an unsinged-meat control to refine this distinction. Despite these limitations, our results provide compelling evidence that non-standard singeing fuels—particularly used tyres—pose significant health risks. These findings highlight reproductive hormones as valuable indicators of endocrine and systemic disruption, without implying temporal precedence, and underscore both the health risks of unsafe singeing practices and their broader evolutionary implications for fertility and population fitness.

## Figures and Tables

**Table 1:** Summary of clinical observations and endpoint criteria applied during the 12-week feeding study in male Wistar rats.

Category	Parameters Evaluated
General appearance & behavior	Posture, coat condition, grooming, locomotor activity, responsiveness
Food & water intake	Daily monitoring for changes in appetite or consumption
Body weight	Recorded weekly throughout the 12-week feeding period
Clinical signs of toxicity	Piloerection, salivation, respiratory distress, tremors, abnormal gait, lethargy, diarrhea
Endpoint criteria	>20% body weight loss, persistent inability to eat or drink, severe respiratory or neurological distress, moribund condition

## Data Availability

Data sharing is not applicable to this study.
